# An Unusual Cardiac Manifestation in Autosomal Dominant Polycystic Kidney Disease

**DOI:** 10.1155/2012/978170

**Published:** 2012-11-22

**Authors:** Fausta Catapano, Stefano Pancaldi, Carlo Pace Napoleone, Lucia Barbara De Sanctis, Gaetano Gargiulo, Giuseppe Emiliani, Antonio Santoro

**Affiliations:** ^1^Division of Nephrology, Dialysis and Hypertension, Policlinico S. Orsola-Malpighi, 40138 Bologna, Italy; ^2^Cardiology Unit, Policlinico S. Orsola-Malpighi, 40138 Bologna, Italy; ^3^Pediatric Cardiac Surgery Unit, Policlinico S. Orsola-Malpighi, 40138 Bologna, Italy; ^4^Nephrology and Dialysis Unit, Ospedale Santa Maria delle Croci, 48121 Ravenna, Italy

## Abstract

Autosomal dominant polycystic kidney disease is a common hereditary disorder characterized by renal and extrarenal, cystic and noncystic manifestations. Connective tissue defects, including cerebral aneurysm, meningeal diverticula, abdominal wall hernias, intestinal diverticula, and cardiac valvular abnormalities, are widely known manifestations. Instead intracardiac aneurysms have never been reported in adults with autosomal dominant polycystic kidney disease. We describe a 65-year-old patient with end-stage renal disease due to autosomal dominant polycystic kidney disease and an atrial septum aneurysm associated with platypnoea-orthodeoxia syndrome.

## 1. Introduction

Autosomal dominant polycystic kidney disease (ADPKD) is the most common inherited renal disease and occurs in 1 in 400–1,000 individuals [1]. ADPKD is genetically heterogeneous; two genes—*PKD1 *and *PKD2*—are implicated in its development [1].

The disease is characterized by renal and extrarenal involvement with cystic and noncystic manifestations. There are well-recognized extrarenal noncystic associations such as gastrointestinal tract diverticula, cerebral aneurysm, hypertension, and cardiovascular abnormalities. Hypertension is a common early finding of ADPKD and occurs in approximately 60% of patients prior to renal function impairment [2, 3]. Left ventricular hypertrophy also occurs in patients with ADPKD [4]. Moreover, other specific cardiac abnormalities, such as valvular anomalies, have been described in this disease.

In this report, we describe a patient with end-stage renal disease (ESRD) secondary to ADPKD who presented dyspnoea induced by upright position and relieved by recumbence configuring the platypnoea-orthodeoxia syndrome (POS). A transesophageal echocardiography (TEE) showed an aneurysm of atrial septum (ASA) with patent foramen ovale and right-to-left shunt. A significant dilatation of ascendant aorta was also found.

## 2. Case Report

We report the case of a 65-year old patient with a long-standing hypertension and a *family history* of ADPKD. In 1996, he presented an embolic transient ischemic attack, and ASA was found with no other abnormalities on two-dimensional echocardiogram. Abdomen ultrasound also showed multiple kidney and hepatic cysts. In 2001, after spontaneous rupture of a cyst with massive haemorrhage, the left kidney was removed and chronic kidney disease diagnosed. In 2002 he suffered from thoracic pain and severe asthenia; the exercise test stress excluded ischaemia. In 2008 serum creatinine started increasing and an arteriovenous fistula was created.

**Figure 1:**
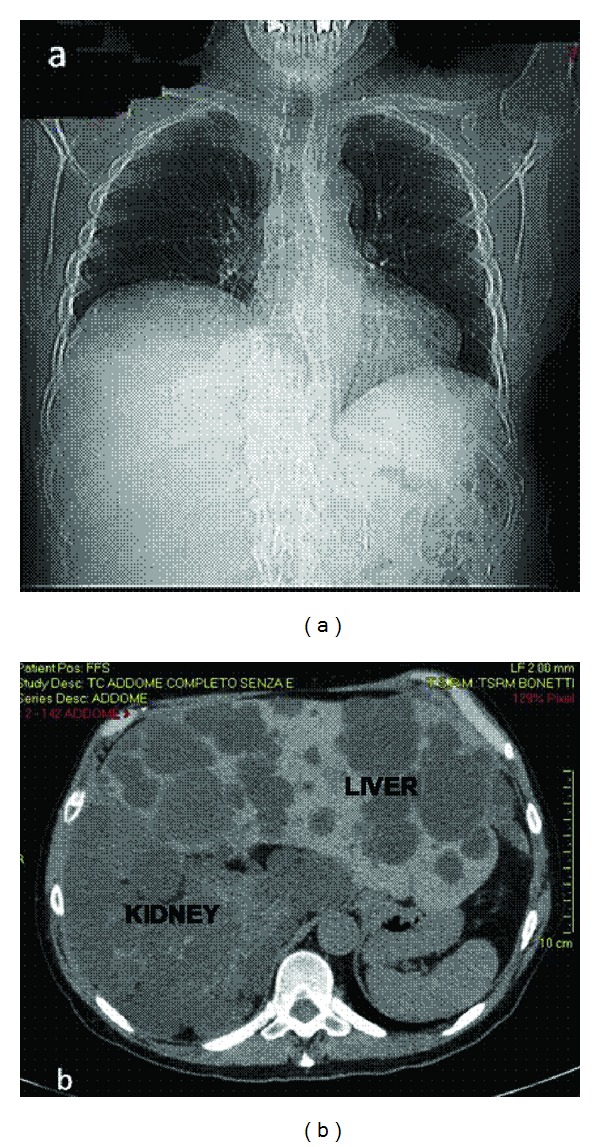
(a) Chest X-ray showing the elevation of the right diaphragm; (b) abdomen multislices CT-scan view showing the hepatic and kidney cysts.

On January 29, 2010, he was admitted to a local hospital for severe dyspnoea induced by upright position and relieved by recumbence. At chest X-ray, heart, and lungs appeared normal, and an elevation of the right diaphragm, as a result of the encumbrance of the right kidney and hepatic cysts, was seen (Figure 1(a)).

Abdomen multislices CT-scan showed an enormous and single right kidney with multiple cysts of diameter between 5 and 7 cm; and a polycystic liver with the biggest cyst, in right lobe, measuring 12 cm (Figure 1(b)).

Further investigation showed a restrictive pulmonary function deficit due to the mechanical compression of the right lung (total lung capacity (TLC) 4.79 L (79% pred), vital capacity (VC) 2.57 L (72% pred), forced expiratory volume in second (FEV_1_) 1.81 L (67% pred)) with no alterations in the diffusing capacity of the lungs for carbon monoxide. Pulmonary embolism was excluded by pulmonary angiography. Left ventricular hypertrophy (LVmass, 128 g/m^2^) was present on two-dimensional echocardiogram, and the diagnosis of ASA with no shunt evidence was confirmed. Ejection fraction and pulmonary artery pressure (27 mmHg) were normal in supine position. A dilation of aortic root (46 mm), a mild aortic and mitral incompetence, was also diagnosed.

Upon laboratory analysis, cardiac enzymes were found to be normal, while serum creatinine and blood nitrogen urea were 708 umol/L and 26.67 mmol/L (8 and 160 mg/dL), respectively. The nephrologists of the local hospital decided to submit the patient to haemodialysis session. The session was early interrupted owing to severe oxygen desaturation and hypotension; then the patient was transferred to our Nephrology Unit.

Upon admission urinary output was preserved. In upright position the patient presented severe dyspnoea, dizziness, and a sense of fainting so that he was forced to lie down in bed. On physical examination there was a large palpable liver and a right kidney extending to right iliac fossa. Blood pressure and cardiac output dropped in upright position, whereas heart rate rose when the patient stood up (Table 1). The arterial blood gas analysis showed a severe hypoxemia in upright position, whereas in supine position, the gas-analysis picture normalized (Table 1). These findings suggested the diagnosis of platypnoea-orthodeoxia syndrome in the hypothesis of a right-to-left shunting.

**Figure 2:**
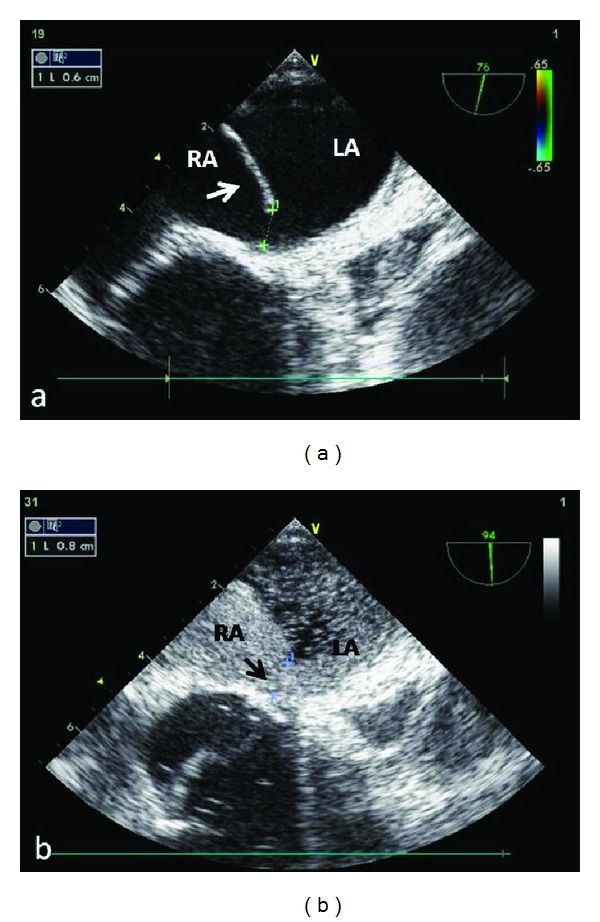
Transesophageal echocardiogram demonstrating: (a) the aneurysm of the atrial septum (white arrow) and (b) the size of patent foramen ovale (0.8 cm) with right-to-left shunting (black arrow) after agitated saline contrast injection. RA, right atrium; LA, left atrium.

Therefore transesophageal echocardiogram (TEE) was performed in supine as well as in sitting position. In the latter position, ASA with a large patent foramen ovale could be observed (Figure 2(a)). A significant right-to-left shunt was confirmed after the injection of an agitated saline contrast solution in the right atrium (Figure 2(b)).

He was thus addressed to heart surgery and submitted to surgery. After median sternotomy cardiopulmonary bypass was instituted. In cardioplegic arrest, a right atriotomy disclosed a widely patent foramen ovale with a huge aneurysm of the interatrial septum and a redundant Eustachian valve. The spatial relationship between the inferior vena cava and these structures caused a selective right-to-left shunt of the blood coming from the inferior vena cava. The aneurysm was resected and the foramen ovale was closed with running suture. The day after surgery the platypnoea and orthodeoxia were completely resolved (Table 1). The postoperative stay was uneventful, and after five days the patient was discharged in a good condition.

## 3. Discussion

We report an unusual cardiac manifestation in ADPKD: interatrial right-to-left shunting through an atrial septal defect in the absence of pulmonary hypertension, expressing platypnoea-orthodeoxia syndrome.

Platypnoea is defined as dyspnoea induced by upright posture and relieved by recumbence; orthodeoxia is arterial deoxygenation accentuated by upright position and improved by recumbency. Several mechanisms of right-to-left atrial shunting have been reported: altered right heart compliance, anatomical alterations of the right atrium, such as fossa ovalis distortion [5, 6], mechanical compression by hydrothorax, or right hemidiaphragm elevation due to pulmonary lobectomy. These anatomical anomalies may cause distortion of atrial septum and lead to foramen ovale patency.

In our patient an increase of the intrathoracic pressure may have been the cause of the shunt. The mechanical compression of the right atrium between the dilated aorta and the huge intra-abdominal mass was responsible, in presence of an aneurysmatic atrial septum, for the right-to-left shunt through the patent foramen ovale. However, at the time of the first echocardiogram the shunt had not been found. At that time the abdominal mass and the aortic bulb were smaller and therefore exerted less pressure on the right atrium. Also, only transthoracic echocardiogram was performed, and this is not the best technique to highlight foramen ovale. In the past history of the patient the occurrence of a transient ischemic attack should have raised suspicion of a paradox embolism through a patent foramen ovale [5–7].

ASA has never been described among the copathologies of adult ADPKD. There is only a report by Waz et al., which describes a 6-year-old patient with ADPKD, normal renal function, hypertension, and serial echocardiograms showing an aneurysm of the atrial septum [8]. Korzets et al. described a case of a 72-year-old Caucasian female with ESRD due to ADPKD, who presented shortness of breath markedly exacerbated by standing up and alleviated by lying down. On echocardiogram, normal-sized chambers were visualized, with good left ventricular contractility. The authors, to account for this phenomenon, advanced the hypothesis of the existence of intrapulmonary shunting associated with intrapulmonary vascular dilatation. This is the same mechanism called to explain severe hypoxemia in chronic liver diseases [9]. However, in this case it is not possible to exclude a right-to-left interatrial shunt through a misdiagnosed patent foramen ovale.

The pathogenesis of intracardiac aneurisms in ADPKD is uncertain. It may reflect a defect in the structure and function of connective tissue and extracellular matrix [10]. In some families with ADPKD, connective tissue and/or extracellular matrix defects may contribute to skeletal abnormalities such as pectus abnormalities, pes planus, joint laxity, arachnodactyly, scoliosis, dolichostenomelia, and high arched palate [11]. These abnormalities are reproduced by targeted mutations of the *Pkd1* gene in mice [12, 13]. Moreover, Wu et al. have demonstrated that polycystin-2 is essential to normal development of the interventricular and interatria septa [14]. In fact, *Pkd2* ^−/−^ mice die *in utero* between embryonic day and parturition; they have structural defects in cardiac septation and cyst formation in maturing nephrons [14]. Finally, various connective tissue dysplasias have also been associated with cystic kidneys in humans [15]. A similar disorder in collagen metabolism has been noted for the Ehlers-Danlos (type I and III) and Marfan syndromes. In our patient this inheritable abnormality of collagen metabolism could explain the development of atrial aneurysm, which was the *Primum movens* in the genesis of the platypnoea-orthodeoxia syndrome.

As previously mentioned, an elevation of diaphragm eventually associated with ascendant aortic dilatation is the cause of patent foramen ovale and platypnoea-orthodeoxia syndrome. In ADPKD patients this mechanism is due to the abdominal mass represented by the huge hepatic and kidney cysts.

In conclusion, atrial septal aneurysm is a rare finding in ADPKD patients. Transthoracic echocardiogram can make diagnosis; however, transesophageal echocardiogram is the gold standard to detect the existence of interatrial communication. Furthermore, if the patient suffers from severe dyspnoea induced by the upright position, TEE (better with saline contrast study) should be repeated in the upright position to diagnose patent foramen ovale with right-to-left shunting. Increased awareness of this extrarenal manifestations in patients with ADPKD may lead to its early diagnosis and treatment.

## Figures and Tables

**Table 1:** Hemodynamic parameters and arterial pO_2_ in different positions before and after surgery.

Parameter	Before surgery		After surgery	
	Supine	Upright	Supine	Upright
BP (mmHg)	117/73	103/84	130/80	120/70
HR (bpm)	98	112	87	98
Cardiac output (L/min)	5.0	3.6	5.2	4.9
SaO_2_ (%)	85	66	94	93
PaO_2 _(mmHg) breathing room air	79.6	32.8	106	90
